# Supplementary material for: Intra-gastric phytoliths provide evidence for folivory in basal avialans of the Early Cretaceous Jehol Biota
Source: Nat Commun. 2023 Jul 28;14:4558. doi: 10.1038/s41467-023-40311-z (PMC10382595; doi:10.1038/s41467-023-40311-z)
Supplement: Supplementary file 1 — Supplementary Information [file 41467_2023_40311_MOESM1_ESM.pdf]

**Supplementary Information for**

# Intra-gastric phytoliths provide evidence for folivory in basal avialans of the Early Cretaceous Jehol Biota

Yan Wu et al.

**This PDF file includes:**

Supplementary Text
Supplementary Figures 1-7
Supplementary Tables 1-2
Supplementary references

**Supplementary Note 1：Systematic Paleontology**

*Jeholornis* Zhou & Zhang, 2002

Type species *Jeholornis prima* Zhou & Zhang, 2002

The new specimen (IVPP V14978) was acquired in Gong-Gao Village in Chaoyang County in western Liaoning Province, northeastern China during the 2006 field season led by IVPP crews, and it was deposited in the collection of the Institute of Vertebrate Paleontology and Paleoanthropology in Beijing. It can be referred to *Jeholornis prima* by the combination of both skeletal and integumental morphologies[1,2].

As in the holotype of *J. prima* and other referred specimens of *Jeholornis*, the overall shape of the diapsid skull in the new specimen is triangular in lateral view (Fig. 1). Its rostrum is largely edentulous with only one or two tiny dentary teeth and a maxillary tooth recognizable (Supplementary Fig. 1 and Supplementary Dataset 1). The premaxillae bear a dome-like arch with the indented junction close to rostral end of the nasal (Supplementary Fig. 1). The wide rod-like maxillae occupy a large portion of the skull, which is about one-third of the entire skull length. The lower jaw is robust as in other individuals of Jeholornithidae[3]. The rostro-dorsal trend of the dentary is slightly convex in the middle as previously seen in the holotype and other referred specimens of *J. prima*, in addition to other closely related taxa[4,5]. The tetraradiate palatine bone is exposed under the maxillae (Supplementary Fig. 1). Differing from *Archaeopteryx* and other non-avialan theropods, the caudal extension of the palatine of the new specimen is shorter. Caudally, a nearly square-shaped squamosal is recognized, with its dorsal and ventral margins slightly concave. The thick rod-like jugal bar bears a large dorsal process caudally. An inverted ‘L’-shaped quadratojugal is present, caudal to the quadratojugal (Supplementary Figs. 1 and 2). The outline of the quadrate forms a right triangular shape in lateral view with the hypotenuse as the rostral margin (Supplementary Fig. 1). Under the postdentary, the hyoid apparatus is composed of a separate bony ceratobranchial and dorsally deflected epibranchial elements (Fig. 1 and Supplementary Fig. 2).

The robust furcula is boomerang-like, and preserved underneath other cervical bones, being visible only in the CL slices (Supplementary Dataset 1). The strut-like coracoid bears a limited procoracoid process and an oval-shaped supracoracoideus nerve foramen under the blunt acrocoracoid process (Supplementary Fig. 2). The procoracoid process extends medially into a flange and distally, a process is present along its lateral margin in distal end. The lateral margin of coracoid is slightly convex (Supplementary Fig. 1).

The unfinished appearance of humerus (proximal periosteal part) suggests the bird individual died in at a subadult stage of development. The primary and secondary pennaceous feathers are well developed and long, which articulate with the forearm forming a very pointed wing shape (Fig. 1). The manual digits of *Jeholornis* are quite distinct, and the phalanges of digits II and III are identified with articulated distal unguals in the new specimen. The manual ungual of the major digit (digit II) is larger than that of digit III (Supplementary Fig. 1). The unreduced manual phalanx of digit III (III-3) is long and slender as in the holotype of *Jeholornis prima*. The robust manual phalanx II-2 is longer than the phalanx III-3 (Supplementary Fig. 1). This difference is a feature that can be used to distinguish *Jeholornis* from other more basal avialan and other more derived ornithuromorphs[2,4].

The femur of the new specimen is only slightly longer than that of another referred specimen of *Jeholornis prima* (IVPP V 13350)[2]. The distal tibiotarsus has an astragalar ascending process. The metatarsal II is the shortest of the three metatarsals with metatarsal IV slightly longer, and metatarsal III the longest (Supplementary Fig. 1). The relative extension of three metatarsals distally distinguishes the new specimen from that of *J. palmapenis*, in which the metatarsi trochlea II and IV are rather close to each other in their distal extension[4]. The second metatarsal is slightly deflected medially. Other features the distinguish the two species from one another include the presence of a proximal fan-shaped feather cluster above the pelvis in *J. palmapenis*, that is absent in *J. prima* (and the new specimen).

The synsarcral vertebrae include five to six fused vertebrae with long transverse processes identified in the caudal two fused vertebrae (Supplementary Fig. 2). The dorsal surface of the ilium appears to be flat, differing from the convex dorsal margin in *J. palmapenis.* The caudal part of the ilium tapers and bends ventrally. The ischium has a dorsal process that projected high with a relatively lower intermediate process present in other *Jeholornis*[2] specimens**.** The intermediate process of the ischium is located near the middle part of the ischial shaft. The distal pubis bears an expanded ball-like process.

At least 23 caudal vertebrae are recognizable and comprise the long bony tail, consistent with the published range for Jeholornithidae[3]. The cranial portion of the caudal vertebrae are shorter than the middle ones, forming a wide and square shape. The caudal vertebrae increase in length with the longest ones being the 5$^{th}$ -10$^{th}$ caudals Caudally, they then become gradually tapered and narrower dorsoventrally (about 10$^{th}$ - 22$^{nd}$). The bony chevron series changes its shape as well along with the transition to the different caudal regions. The chevron bones are well developed in the cranial ones of the caudal vertebrate (up to 4$^{th}$ -5$^{th}$), and they have rectangular shape., Then, they become slim and rod-like shape in the middle and caudal region of the caudal vertebrae. The pre- and post-zygapophyses within the long-shaped middle caudal series is fork-like, and they are tightly interlinked together.

The distal caudal vertebrae of the new specimen are articulated with the small frond-like rectrices in the last five caudal vertebrae (Supplementary Fig. 1). In total, at least six pennaceous feathers are recognizable on each side (Supplementary Fig. 1). The morphology of the tail feathering is like that of the referred specimen of *Jeholornis* (IVPP V13350), which is also associated with the distal six caudal vertebrae, forming a small rectrical fan. As previously argued, the small rectrices plane formed by these tail feathers had limited aerodynamic function, and they might only have served a sexual or ornament signaling role[6,7].

This dentary (rostro-dorsal trending) morphology is comparable to the that of the extant hoatzin (*Opisthocomus hoazin*), whose main diet are swamp tree leaves in tropical forests[8]. The feature was identified as indirect and supportive evidence of definite plant-based feeding in birds[9]. As shown in the revaluation of GMM data, many selected taxa of herbivorous Galliformes and Anseriformes, share a similar mandibular shape with the hoatzin and the *Jeholornis*. Two raptorial birds (*Vultur* and *Micrastur*) also plot quite close to *Jeholornis*, and this position could be the result of convergence in jaw function for tearing food items shared by these taxa. For the hoatzin, the unreduced manual unguals in the juvenile hoatzin resemble those of *Jeholornis*, in terms of functioning in arboreal climbing[10].

The size of the specimen also is consistent with this ontogenetic assessment, which is smaller than the holotype, but close to a smallest referred specimen (IVPP V13350). A histological section taken from the rib segment documents only one line of arrested growth line. The osteo-histological and other rough surface features on bone support our assessment of its subadult stage.

**Supplementary Note 2: Identification of the Stomach content**

The stomach content is identified as carbonized materials mostly concentrated within the thoracic rib cage. The identifiable stomach content is determined by their position, black color, and woods-like text which likely formed with the carbonatization of organic materials during diagenesis. The residue is specifically located within the thorax cavity trapped within in the abdominal part of the body within the rib cage. A few gastroliths identified with a smooth surface and oval shape are clumped within the gut content residues (Supplementary Dataset 1). The packing and arrangement of this residue support it belonging to the stomach and was separated away from the bottom-matrix (Supplementary Fig. 1).

**Supplementary Figures**

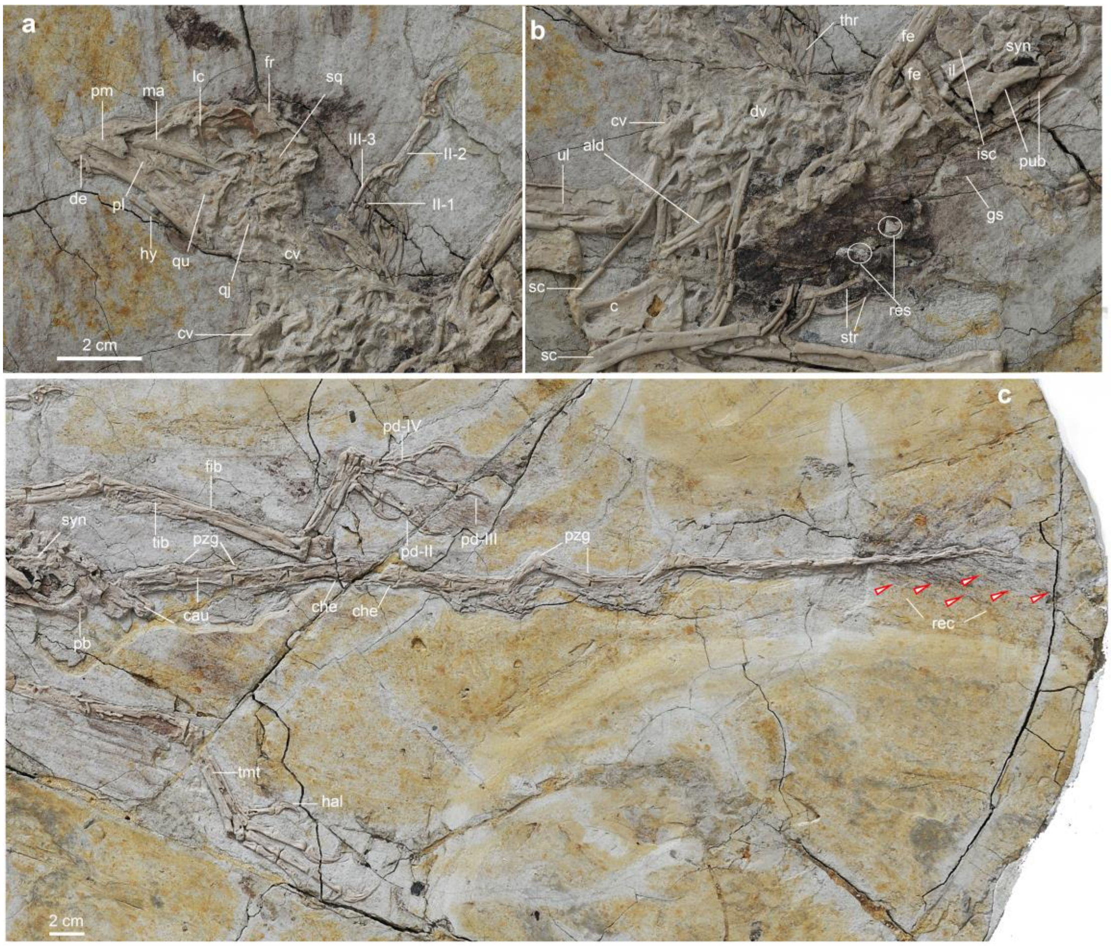


**Supplementary Figure 1.** The enlarged photos with detailed anatomical features revealed in the new referred *Jeholornis* specimen (IVPP V14978). (a) cranial part, (b) thoracic part, (c) caudal part. Anatomical abbreviations: ald, alular digit; c, coracoid; cau, caudal vertebrate; che, chevron; cv, cervical vertebrate; de, dentary; dv, dorsal vertebrate; fe, femur; fib, fibula; hal, hallucal claw; fr, frontal; hy, hyoid; II-1, phalanx II-1; II-2, phalanx II-2; III-3, phalanx III-3; il, ilium; isc, ischium; lc, lacrimal; ma, maxilla; pb, pubic boot; pd-II, III, and IV, pedal digits II, III, and IV; pl palatine; pm, premaxilla; pzg, pre- and post- zygopophysis of the caudal vertebrae; qj, quadratojugal; qu, quadrate; rec, rectrix; res, residue of the stomach content extracted; sc, scapula; sq, squamosal; syn, synsacrum; thr, thoracic rib; tib, tibiotarsus; and tmt, tarsometatarsus.

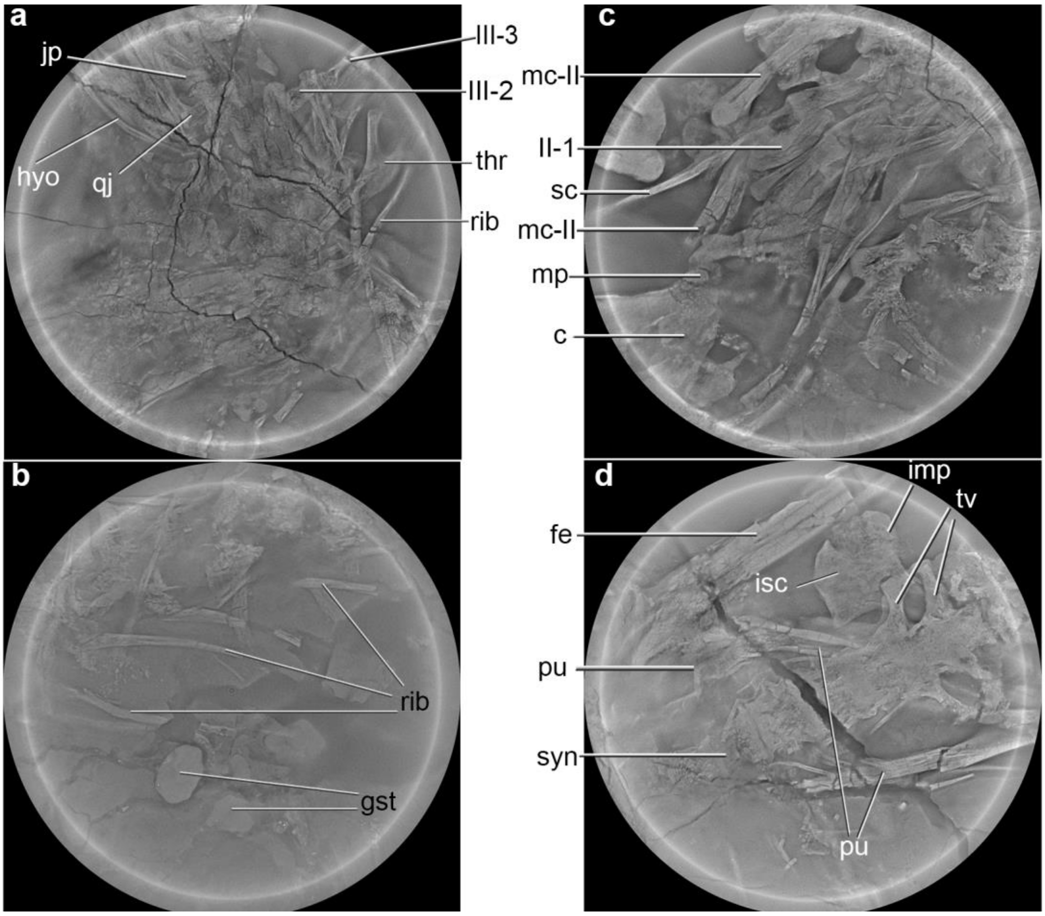


**Supplementary Figure 2.** CL scan slices with a focus of different regions imaged for the new specimen, (a) caudal skull, (b) thoracic region, (c) cervical and pectoral region, (d) pelvic region. Anatomical abbreviations: c, coracoid; fe, femur; gst, gastroliths; hyo, hyoid; II-1, phalanx II-1; II-2, phalanx II-2; III-3, phalanx III-3; imp, intermedial process of ischium; jp, jugal process; isc, ischium; mc-II, major metacarpal; mp, medial process of coracoid; pu, pubis; qj, quadratojugal; sc, scapula; syn, synsacrum; and tv, transverse process.

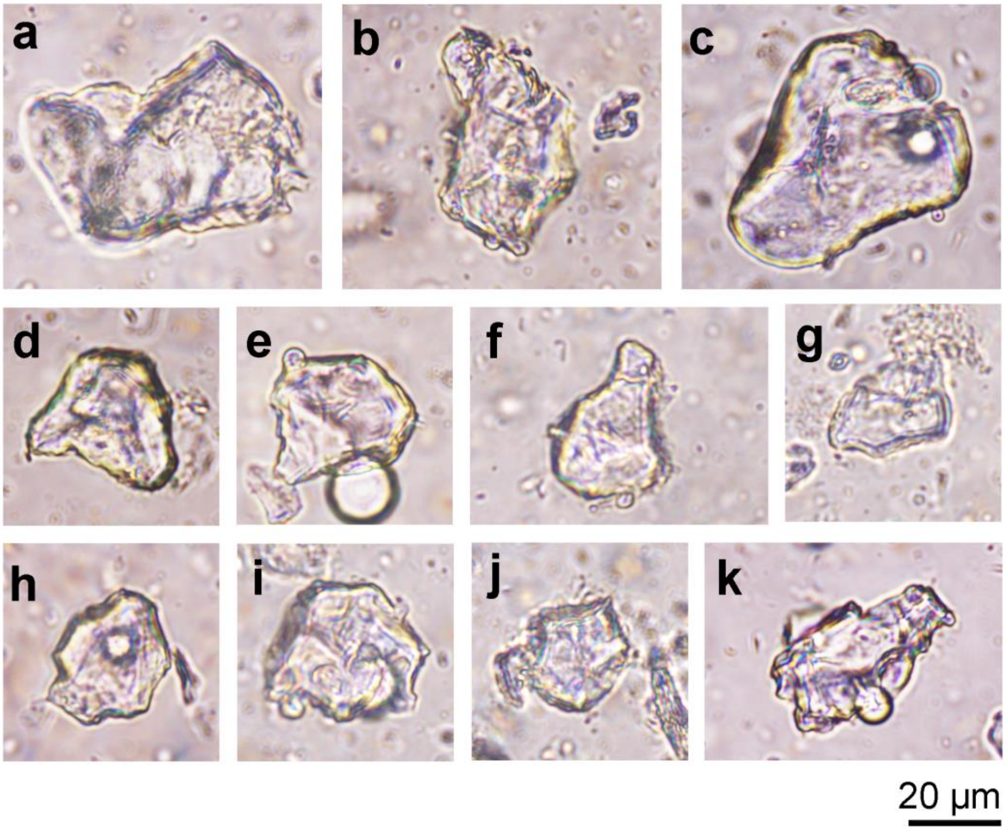


**Supplementary Figure 3**. Phytoliths extracted from stomach content of the referred specimen of *Jeholornis* (IVPP V14978). (a) to (j) was identified as the blocky phytoliths; (k) is an unidentifiable phytolith. Independent experiments were performed twice with similar results; scale bar applies to all panels.

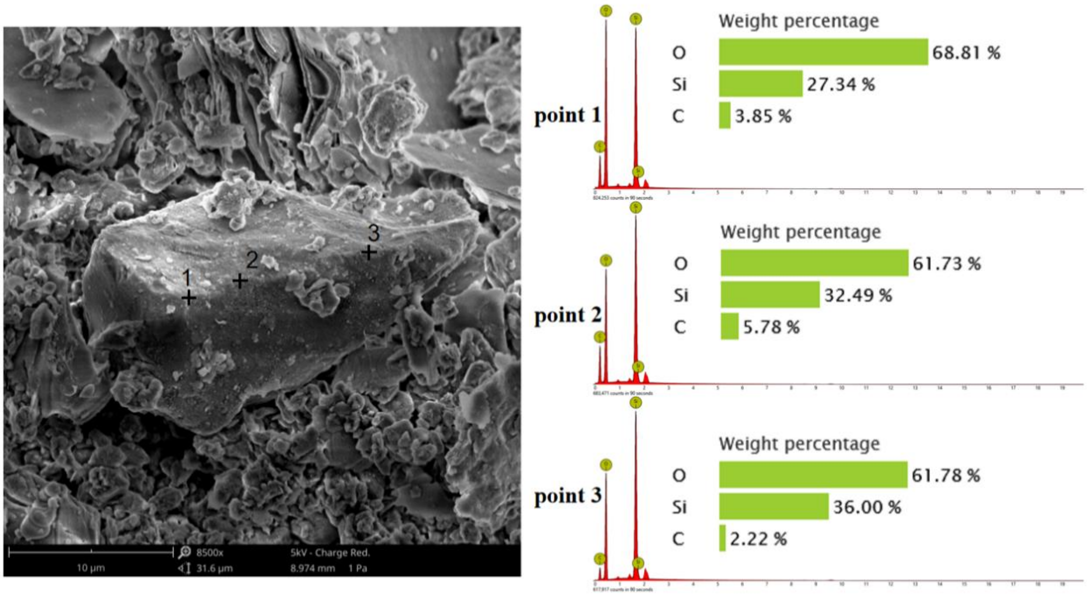


**Supplementary Figure 4.** SEM-EDX analysis of a phytolith recovered from the stomach content of the new *Jeholornis* specimen.

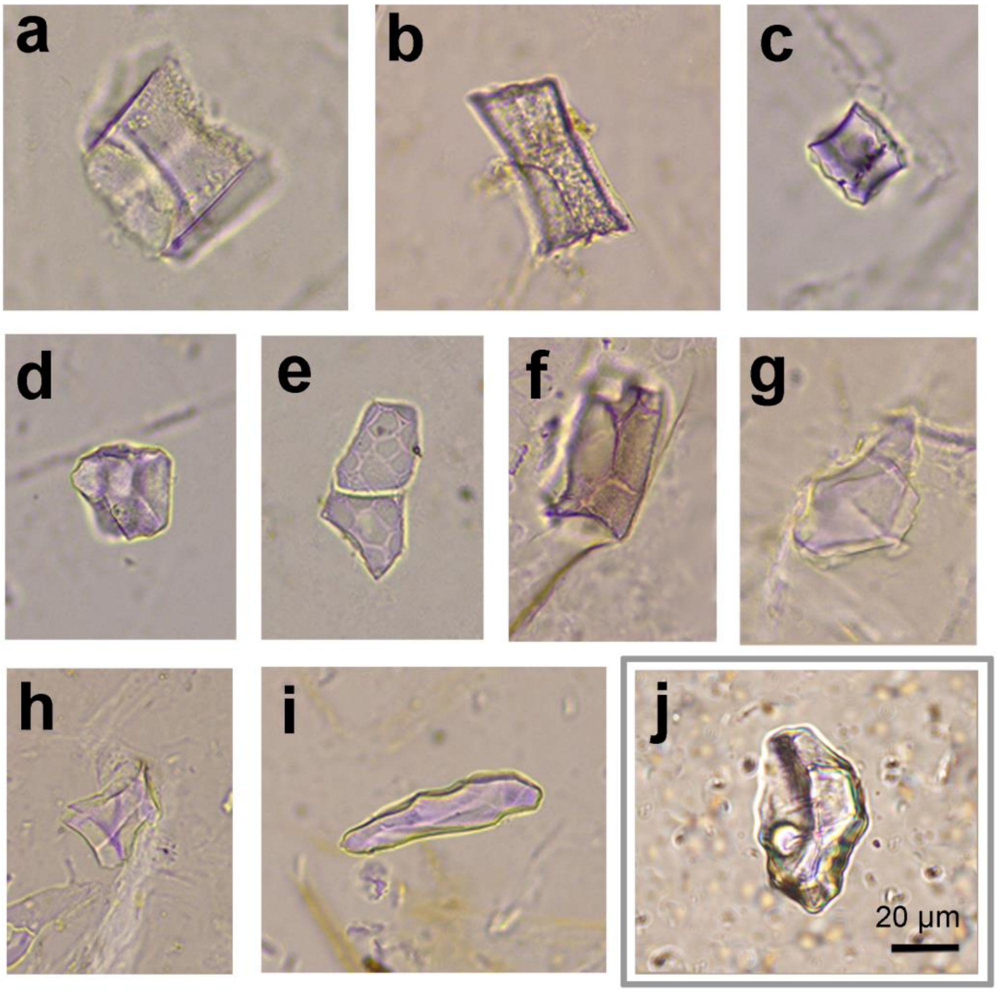


**Supplementary Figure 5.** Comparison between blocky phytoliths with the ridgeline ornament as proposed the modern candidates (a-i) to which the fossil (j) likely relates. (a) *Equisetum ramosissimum* from Yunnan, China; (b) *Pinus armandii* from Tibet, China; (c)-(d) *Oryza sativa* from Zhejiang, China; (e) *Quercus* sp. from Sichuan, China; (f) *Magnolia* sp. from Luzon, Philippines; (g), (i) *Machilus nanmu* from Sichuan, China; (j) fossil blocky phytoliths with wavy ridgelines from the stomach content of *Jeholornis* (IVPP V14978), possibly related to the blocky phytoliths in modern Magnoliales leaves (f) to (i). Independent experiments were performed twice with similar results; scale bar applies to all panels.

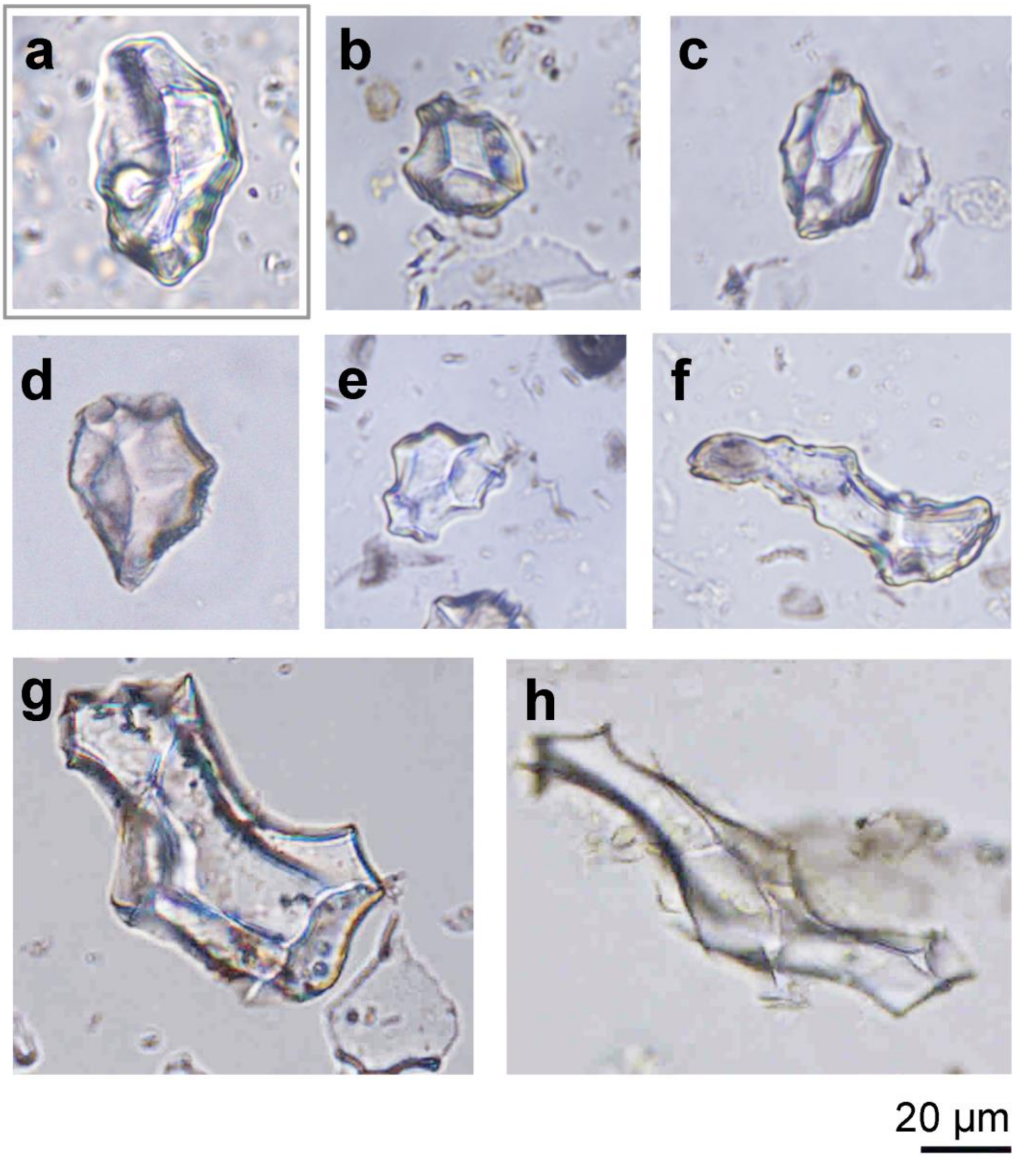


**Supplementary Figure 6.** Comparison between the blocky fossil phytoliths extracted from the digestive tract content of *Jeholornis* (IVPP V14978) **(a)** and blocky phytoliths with the ridgeline ornament extracted from modern Magnoliaceae **(b-h)**.

(a), fossil blocky phytoliths with wavy ridgelines from the stomach content of *Jeholornis* (IVPP V14978), possibly related to blocky phytoliths in modern Magnoliales leaves. (b), *Lirianthe henryi* (Dunn) N.H. Xia & C.Y. Wu; (c), *Magnolia coco* (Lour.) DC. (d), *Manglietia decidua* Q.Y. Zheng; (e), *Manglietia fordiana* Oliv.; (f), *Yulania denudata* (Desr.) D.L. Fu; (g), *Magnolia championii* Benth. (h), *Yulania amoena* (W.C. Cheng) D.L. Fu. Independent experiments were performed twice with similar results; scale bar applies to all panels.

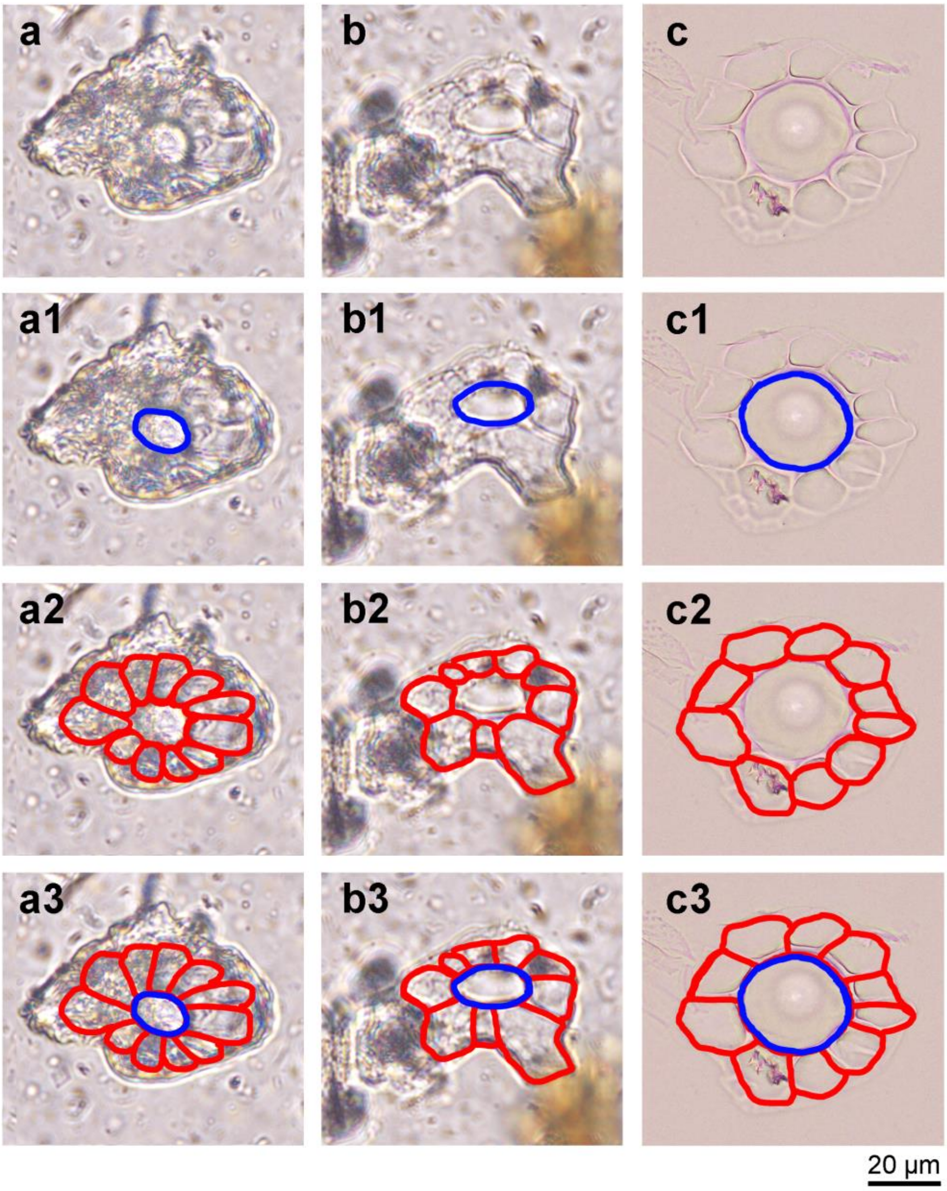


**Supplementary Figure 7.** Comparison between one additional type of fossil phytolith **(a, b)** extracted from the digestive tract content of *Jeholornis* (IVPP V14978), which has a round shape with a protuberant center, with similar modern hair base phytoliths extracted from extant *Ficus tikoua* leaves **(c).** a1-a3, b1-b3, and c1-c3 are respective images of a, b, c with highlighted key features in blue and red. Blue outlines indicate the center papillae of the hair base. Red highlights the surrounding cells whose cell walls formed the surrounding radiate lines. These two key characteristics identified in the fossil hair base are consistent with the modern hair base. Scale bar applies to all panels.

**Supplementary Table 1.** Measurements of major elements in referred specimen of *Jeholornis* IVPP V14978 (in mm).

| Elements | Right side | Left side | | Ratio |
|---|---|---|---|---|
| Humerus length | 87.1 | 90.0 | Humerus/ femur length | 1.62 |
| Ulna length | 87.9 | | Ulna/Humeral length | 0.99 |
| Femur length | 55.2 | 54.3 | Tibiotarsus/ femur length | 1.26 |
| Tibiotarsus length | 67.2 | 70.5 | | |
| Tarsometatarsi III length | 33.0 | 37.0 | | |
| Skull length | 53.3 | | | |
| Coracoid height | 27.2 | | | |

**Supplementary Table 2.** Sampled bird lists with dietary survey used in the GMM plots.

| R_name | Diet | Genus |
|---|---|---|
| *Acanthisitta_chloris*_YPM_125219 | Other | *Acanthisitta* |
| *Accipiter_nisus*_NHMUK_S1982.149.1 | Other | *Accipiter* |
| *Alcedo_atthis*_NHMUK_S_1994.7.1 | Other | *Alcedo* |
| *Anas_discors*_NHMUK_S2001.41.5 | Other | *Anas* |
| *Anhinga_anhinga*_FMNH_491374 | Other | *Anhinga* |
| *Anser_fabalis*_NHMUK_1895.2.6.9_ | Plant eaters (Anseriformes) | *Anser* |
| *Anseranas semipalmata* NHMUK 1852.7.22.1 | Plant eaters (Anseriformes) | *Anseranas* |
| Apaloderma_narino_FMNH_290897_ | Other | *Apaloderma* |
| Apteryx_australis_FMNH_391011 | Other | *Apteryx* |
| Aramus_guarauna_FMNH_376078 | Other | *Aramus* |
| Archilochus_colubris_FMNH_484738 | Other | *Archilochus* |
| Ardea_alba_UMZC_338H | Other | *Ardea* |
| Arenaria_interpres_FMNH_313992 | Other | *Arenaria* |
| Aythya_ferina_NMHUK_1851.12.23.15 | Plant eaters (Anseriformes) | *Aythya* |
| Balaeniceps_rex_NHMUK_S1952.1.100 | Other | *Balaeniceps* |
| Balearica_pavonina_NHMUK_1859.10.26.5_ROGER | Other | *Balearica* |
| Barnardius_zonarius_NHMUK_S_1983.5.1 | Other | *Barnardius* |
| Bonasa_umbellus_FMNH_348972 | Plant eaters (Galliformes) | *Bonasa* |
| Bucco_capensis_FMNH_330305 | Other | *Bucco* |

| | | |
|---|---|---|
| Bucorvus_abyssinicus_NHMUK_S2006.31.20_ | Other | *Bucorvus* |
| Burhinus_senegalensis_FMNH_313704 | Other | *Burhinus* |
| Buteo_rufofuscus_NHMUK_1862.1.18.1 | Other | *Buteo* |
| Calandrella_cinerea_NHMUK_S1961.13.34 | Other | *Calandrella* |
| Calyptomena_viridis_NHMUK_s1969.1.70 | Fruit | *Calyptomena* |
| Capito_niger_FMNH_321049 | Fruit | *Capito* |
| Caprimulgus_macrurus_FMNH_392245 | Other | *Caprimulgus* |
| Caracara_cheriway_UMMZ_133664 | Other | *Caracara* |
| Cariama_cristata_FMNH_105653 | Other | *Cariama* |
| Cathartes_burrovianus_UMMZ_227466 | Other | *Cathartes* |
| Chaetura_brachyrua_UMMZ_157689 | Other | *Chaetura* |
| Charadris_vociferus_FMNH_470173 | Other | *Charadris* |
| Chauna_chavaria_OUMNH_23790 | Plant eaters (Anseriformes) | *Chauna* |
| Chelidoptera_tenebrosa_YPM_114404 | Other | *Chelidoptera* |
| Chloropipio_holochlora_FMNH_288169 | Other | *Chloropipio* |
| Choloroceryle_amazona_UMMZ_209261 | Other | *Choloroceryle* |
| Chordeiles_minor_UMMZ_233923 | Other | *Chordeiles* |
| Choriotis_kori_UMMZ_210444 | Other | *Choriotis* |
| Chunga_burmeisteri_FMNH_106731 | Other | *Chunga* |
| Climacteris_melanura_UMMZ_214303 | Other | *Climacteris* |
| Colinus_virginianus_FMNH_105065 | Plant eaters (Galliformes) | *Colinus* |
| Colius_macrourus_FMNH_368959_ | Fruit | *Urocolius* |
| Colius_striatus_FMNH_384817 | Fruit | *Colius* |
| Columba_palumbus_UMZC_409A_ | Other | *Columba* |
| Columbina_minuta_FMNH_289160 | Other | *Columbina* |
| Coracias_benghalensis_NHMUK_S1987.19.15 | Other | *Coracias* |
| Corvus_brachyrhynchos_FMNH_441515_ | Other | *Corvus* |
| Corythaeota_cristata_FMNH_313066 | Fruit | *Corythaeola* |
| Crax_mitu_FMNH_320386 | Fruit | *Crax* |
| Crypturellus_tataupa_UMMZ_201948 | Other | *Crypturellus* |
| Cuculus_fugax_FMNH_357420 | Other | *Cuculus* |
| Dendrocygna_bicolor_UMMZ_219885_ | Plant eaters (Anseriformes) | *Dendrocygna* |
| Deroptyus_accipitrinus_NHMUK_S2002.14.2 | Other | *Deroptyus* |
| Diomedea_irrorata_NHMUK_S1963.28.4 | Other | *Diomedea* |
| Elaenia_flavogaster_FMNH_394495 | Other | *Elaenia* |
| Elanus_caerulenus_NHMUK_1850.8.15.159 | Other | *Elanus* |
| Eudromia_elegans_UMMZ_156966 | Other | *Eudromia* |
| Eurypyga_helias_FMNH_317341 | Other | *Eurypyga* |
| Falco_sparverius_UMMZ_154452 | Other | *Falco* |
| Falco_sparverius_UMMZ_154462 | Other | *Falco* |

| | | |
|---|---|---|
| Fregata_aquila_NHMUK_1890.11.3.3 | Other | *Fregata* |
| Fringilla_coelebs_OUMNH_24647 | Other | *Fringilla* |
| Galbula_dea_FMNH_376705_ALL_ | Other | *Galbula* |
| Gavia_stellata_NHMUK 1891.7.20.132 | Other | *Gavia* |
| Glareola_pratincola_FMNH_368875 | Other | *Glareola* |
| Grus_leucogeranus_UMZC_344D | Other | *Grus* |
| Haematopus_ostralegus_FMNH_363899 | Other | *Haematopus* |
| Hemiprocne_comata_UMMZ_158224 | Other | *Hemiprocne* |
| Hirundinea_bellicosa_UMMZ_200850 | Other | *Hirundinea* |
| Hymenops_persipicillata_UMMZ_158790 | Other | *Hymenops* |
| Indicator_exilis_FMNH_429726 | Other | *Indicator* |
| Ixobrychus_minutus_NHMUK_S_2007_61.1_ | Other | *Ixobrychus* |
| Jacana_jacana_NHMUK_S_1990.2.5 | Other | *Jacana* |
| Jynx_torquilla_NHMUK_s1986.36.10 | Other | *Jynx* |
| Larus_novae-hollandiae_UMZC_274.C_ | Other | *Larus* |
| Leptoptilos_crumeniferus_NHMUK_S_1952.3.182 | Other | *Leptoptilos* |
| Leptotila_rufazilla_FMNH_318659 | Other | *Leptotila* |
| Limosa_lapponica_NHMUK_S_1994.46 | Other | *Limosa* |
| Malurus_melanocephalus_UMMZ_224775 | Other | *Malurus* |
| Megalaima_chrysopogon_NHMUK_1850.8.15.28 | Fruit | *Megalaima* |
| Melanopareia_torquata_FMNH_335140 | Other | *Melanopareia* |
| Menura_novaehollandiae_FMNH_336751 | Other | *Menura* |
| Micrastur_ruficollis_FMNH_330226 | Other | *Micrastur* |
| Momotus_momota_NHMUK_S_2105.23 | Other | *Momotus* |
| Monias_benschi_NHMUK_1924.11.29.1 | Other | *Monias* |
| Myiobius_varbatus_FMNH_386812 | Other | *Myiobius* |
| Myrmornis_torquata_YPM_106157 | Other | *Myrmornis* |
| Myrmothera_campanisona_FMNH_322348 | Other | *Myrmothera* |
| Neodrepanis_coruscans_FMNH_363807 | Other | *Neodrepanis* |
| Numida_meleagris_FMNH_390428 | Plant eaters (Galliformes) | *Numida* |
| Nyctibius_griseus_UMMZ_136371 | Other | *Nyctibius* |
| Oceanodroma_leucorhoa_NHMUK_S1953.3.10 | Other | *Oceanodroma* |
| Odontophorous_guttatus_UMMZ_210631 | Plant eaters (Galliformes) | *Odontophorous* |
| Opisthocomus_hoazin_NHMUK_S1961.6.1 | Leaf eaters (hoazin) | *Opisthocomus* |
| Ortalis_ruficauda_UMMZ_155489 | Fruit | *Ortalis* |
| Pandion_haliaetus_FMNH_437336 | Other | *Pandion* |
| Pedionomus_torquatus_NHMUK_A1970.12.1 | Other | *Pedionomus* |
| Pelagodroma_marina_NHMUK_1895.7.1.163_ALL | Other | *Pelagodroma* |
| Pelecanoides_urinatrix_NHMUK_S2006.1.14 | Other | *Pelecanoides* |
| Pelecanus_occidentalis_NHMUK_S1973.66.16 | Other | *Pelecanus* |

| | | |
|---|---|---|
| Phaethon_lepturus_NHMUK_1876.3.16.3 | Other | *Phaethon* |
| Phalacrocorax_albiventer_NHMUK_S_2012.36.1 | Other | *Phalacrocorax* |
| Phoenicopterus_roseus_UMZC_346B_ALL | Other | *Phoenicopterus* |
| Phoeniculus_purpureus_FMNH_368956 | Other | *Phoeniculus* |
| Picus_viridis_NHMUK_NHMUK_S1982.5.1 | Other | *Picus* |
| Pipra_erythrocephala_UMMZ_157210 | Fruit | *Pipra* |
| Piprites_chloris_FMNH_290398 | Other | *Piprites* |
| Podica_senegalensis_UMZC_209A_ | Other | *Podica* |
| Poecile_atricapillis_FMNH_504323 | Other | *Poecile* |
| Porphyrio_poliocephalus_NHMUK_S1952.1.93 | Other | *Porphyrio* |
| Probosciger_aterriumus_NHMUK_S2006.15.5 | Other | *Probosciger* |
| Psittacus_erithacus_NHMUK_S1992.41.60_ | Other | *Psittacus* |
| Psittrichas_fulgidus_YPM_141821 | Fruit | *Psittrichas* |
| Psophia_crepitans_FMNH_105783 | Fruit | *Psophia* |
| Pterocles_quadricinctus FMNH 319937 | Other | *Pterocles* |
| Ptilinopus_lechlancheri_FMNH_358261 | Fruit | *Ptilinopus* |
| Ptilonorhynchus_violaceus_UMMZ_155483 | Other | *Ptilonorhynchus* |
| Puffinus_tenuirostris_NHMUK_1850.8.15.152 | Other | *Puffinus* |
| Pycnonotus_caffer_NHMUK_S1986.75.18_01 | Other | *Pycnonotus* |
| Rallus_limnicola_FMNH_501812 | Other | *Rallus* |
| Recurvirostra_avosetta_NHMUk_S1962.10 | Other | *Recurvirostra* |
| Regulus_ignicapillus_NHMUK_S1952.2.662_ | Other | *Regulus* |
| Rhamphastos_ambiguus_NHMUK_S2002.21 | Fruit | *Rhamphastos* |
| Rhynchocyclus_olivaceus_FMNH_330600 | Other | *Rhynchocyclus* |
| Rollandia_rollandia_UMMZ_156975 | Other | *Rollandia* |
| Rollulus_rouloul_NHMUK_1871.7.20.87 | Other | *Rollulus* |
| Rostratula_benghalensis_FMNH_319933 | Other | *Rostratula* |
| Rupicola_peruviana_UMMZ_119210 | Fruit | *Rupicola* |
| Rynchops_niger_FMNH_376309 | Other | *Rynchops* |
| Sagittarius_serpentarius_NHMUK_1898.5.7.1 | Other | *Sagittarius* |
| Sclerurus_mexicanus_FMNH_321715 | Other | *Sclerurus* |
| Scopus_umbretta_FMNH_313701_ | Other | *Scopus* |
| Spheniscus_humboldti_NHMUK_S_2000.7.1 | Other | *Spheniscus* |
| Sterna_hirundo_NHMUK_NHMUK_S_1975.65.4 | Other | *Sterna* |
| Streptoprocne_zonaris_FMNH_85767 | Other | *Streptoprocne* |
| Strix_aluco_NHMUK_S2001.38 | Other | *Strix* |
| Sula_dactylatra_NHMUK_1890.11.3.10 | Other | *Sula* |
| Sylvia_borin_FMNH_385185 | Other | *Sylvia* |
| Terenura_spodioptila_FMNH_344009 | Other | *Terenura* |
| Tigrisoma_lineatum_FMNH_105528 | Other | *Tigrisoma* |
| Tityra_semifasciata_NHMUK_1891.7.20.15 | Fruit | *Tityra* |
| Tockus_nasutus_NHMUK_S_1989.2.2 | Other | *Tockus* |

| | | |
|---|---|---|
| Todus_mexicanus_FMNH_351174 | Other | *Todus* |
| Topaza_pyra_FMNH_318845 | Other | *Topaza* |
| Treron_capellei_UMMZ_220454 | Fruit | *Treron* |
| Tringa_ochrophus_FMNH_368850 | Other | *Tringa* |
| Trogon_melanurus_FMNH_290496 | Fruit | *Trogon* |
| Turdus_olivater_FMNH_319675 | Other | *Turdus* |
| Turnix_varius_NHMUK_S1952.2.142 | Other | *Turnix* |
| Tyrannus_tyrannus_FMNH_487521 | Other | *Tyrannus* |
| Tyto_alba_NHMUK_S1989.22.1_ | Other | *Tyto* |
| Upupa_epops_FMNH_352821 | Other | *Upupa* |
| Vultur_gryphus_NHMUK_S1955.5.11 | Other | *Vultur* |
| Chloris_chloris_OUMNH_24626 | SeedBeak-Finch | *Chloris* |
| Cornuropsis_carolinensis_UMMZ_68741 | SeedBeak-Parrot | *Conuropsis* |
| Ectopistes_migratorius_UMMZ_alcohol | SeedGas | *Ectopistes* |
| *Geospiza_fulginosa*_NHMUK_A1975.15.25 | SeedBeak-Finch | *Geospiza* |
| *Manucodia_chalybatus*_UMZC_436.K | Fruit | *Manucodia* |
| *ParadoxornisTimalia*_NHM1923.1.20.6_1945.14.1_2 | SeedBeak-Finch | *Paradoxornis* |
| *Spizella_arborea*_NHMUK_1986.60.60_2 | SeedBeak-Finch | *Spizella* |
| *Jeholornis_prima*_STM_3-8 | Jeholornis | *Jeholornis* |

Supplementary refences


1. Zhou Z, Zhang F. A long-tailed, seed-eating bird from the Early Cretaceous of China. *Nature* 418, 405 (2002).
2. Z. Zhou, F. Zhang, Jeholornis compared to Archaeopteryx, with a new understanding of the earliest avian evolution. *Naturwissenschaften* **90**, 220-225 (2003).
3. Wang X*, et al.* A new jeholornithiform exhibits the earliest appearance of the fused sternum and pelvis in the evolution of avialan dinosaurs. *Journal of Asian Earth Sciences* 199, 104401 (2020).
4. U. Lefèvre, D. Hu, F. Escuillié, G. Dyke, P. Godefroit, A new long-tailed basal bird from the Lower Cretaceous of north-eastern China. *Biological Journal of the Linnean Society* **113**, 790-804 (2014).
5. J. K. O'Connor, C. Sun, X. Xu, X. Wang, Z. Zhou, A new species of Jeholornis with complete caudal integument. *Historical Biology* **24**, 29-41 (2012).
6. M. Wang *et al.*, An Early Cretaceous enantiornithine bird with a pintail. *Current Biology* **31**, 4845-4852.e4842 (2021).
7. O'Connor J*, et al.* Unique caudal plumage of Jeholornis and complex tail evolution in early birds. *Proceedings of the National Academy of Sciences* **110**, 17404-17408 (2013).
8. L. P. Korzoun, C. Erard, J.-P. Gasc, F. J. Dzerzhinsky, Adaptations of the hoatzin

(Opisthocomus hoazin) to leaf-eating. Morphological characteristics and functional features of its bill and hyoid apparatus. *Comptes Rendus Biologies* **326**, 75-94 (2003).
9. L.E. Zanno and P.J. Makovicky, Herbivorous ecomorphology and specialization patterns in theropod dinosaur evolution. *Proceedings of the National Academy of Sciences*, **108**, 232-237 (2011).
10. A. Abourachid *et al.*, Hoatzin nestling locomotion: Acquisition of quadrupedal limb coordination in birds. *Science advances* **5**, eaat0787 (2019).
