# Supplementary material for: Intra-gastric phytoliths provide evidence for folivory in basal avialans of the Early Cretaceous Jehol Biota
Source: Nat Commun. 2023 Jul 28;14:4558. doi: 10.1038/s41467-023-40311-z (PMC10382595; doi:10.1038/s41467-023-40311-z)
Supplement: Supplementary file 3 — Description of Additional Supplementary Files [file 41467_2023_40311_MOESM3_ESM.pdf]

Description of Additional Supplementary Files

File Name: Supplementary Data 1
Description: CL scans of the new specimen with a focus of skull and thoracic region
