# Supplementary material for: Intra-gastric phytoliths provide evidence for folivory in basal avialans of the Early Cretaceous Jehol Biota
Source: Nat Commun. 2023 Jul 28;14:4558. doi: 10.1038/s41467-023-40311-z (PMC10382595; doi:10.1038/s41467-023-40311-z)
Supplement: Supplementary file 4 — Supplementary Data 1 [file 41467_2023_40311_MOESM4_ESM.pdf]

Description of Additional Supplementary Information

Computed Laminography dataset of the referred specimen of *Jeholornis prima* (IVPP V 14978); the scans were focused on two regions, one with the gastric region and another with the skull.

Compressed tiff images stacks were uploaded in the open OSF database with the link of https://osf.io/hbxfg/
