# Supplementary material for: Intra-gastric phytoliths provide evidence for folivory in basal avialans of the Early Cretaceous Jehol Biota
Source: Nat Commun. 2023 Jul 28;14:4558. doi: 10.1038/s41467-023-40311-z (PMC10382595; doi:10.1038/s41467-023-40311-z)
Supplement: Supplementary file 5 — Reporting Summary [file 41467_2023_40311_MOESM5_ESM.pdf]

nature portfolio

Corresponding author(s): Li Zhiheng

Last updated by author(s): Jun 27, 2023

Reporting Summary

Nature Portfolio wishes to improve the reproducibility of the work that we publish. This form provides structure for consistency and transparency in reporting. For further information on Nature Portfolio policies, see our Editorial Policies and the Editorial Policy Checklist.

Statistics

For all statistical analyses, confirm that the following items are present in the figure legend, table legend, main text, or Methods section.

n/a	Confirmed	
☐	☒	The exact sample size (*n*) for each experimental group/condition, given as a discrete number and unit of measurement
☐	☒	A statement on whether measurements were taken from distinct samples or whether the same sample was measured repeatedly
☒	☐	The statistical test(s) used AND whether they are one- or two-sided<br>*Only common tests should be described solely by name; describe more complex techniques in the Methods section.*
☒	☐	A description of all covariates tested
☒	☐	A description of any assumptions or corrections, such as tests of normality and adjustment for multiple comparisons
☒	☐	A full description of the statistical parameters including central tendency (e.g. means) or other basic estimates (e.g. regression coefficient) AND variation (e.g. standard deviation) or associated estimates of uncertainty (e.g. confidence intervals)
☒	☐	For null hypothesis testing, the test statistic (e.g. *F*, *t*, *r*) with confidence intervals, effect sizes, degrees of freedom and *P* value noted<br>*Give P values as exact values whenever suitable.*
☒	☐	For Bayesian analysis, information on the choice of priors and Markov chain Monte Carlo settings
☒	☐	For hierarchical and complex designs, identification of the appropriate level for tests and full reporting of outcomes
☒	☐	Estimates of effect sizes (e.g. Cohen's *d*, Pearson's *r*), indicating how they were calculated

Our web collection on statistics for biologists contains articles on many of the points above.

Software and code

Policy information about availability of computer code

Data collection	No software was used to collect the data.
Data analysis	GMM re-analysis were conducted using the function of gpagen() for Generalized Procrustes Analysis , gm.prcomp() for PCA, and plotRefToTarget() for shape variations along PC axes from package 'geomorph' in R.

For manuscripts utilizing custom algorithms or software that are central to the research but not yet described in published literature, software must be made available to editors and reviewers. We strongly encourage code deposition in a community repository (e.g. GitHub). See the Nature Portfolio guidelines for submitting code & software for further information.

Data

Policy information about availability of data

All manuscripts must include a data availability statement. This statement should provide the following information, where applicable:

- Accession codes, unique identifiers, or web links for publicly available datasets
- A description of any restrictions on data availability
- For clinical datasets or third party data, please ensure that the statement adheres to our policy

The major parts of images and all other data pertinent to this research are available in the main text and Supplementary Information. Other related image data can also be obtained from the corresponding author lizhiheng@ivpp.ac.cn

Human research participants

Policy information about studies involving human research participants and Sex and Gender in Research.

Reporting on sex and gender	N/A
Population characteristics	N/A
Recruitment	N/A
Ethics oversight	N/A

Note that full information on the approval of the study protocol must also be provided in the manuscript.

Field-specific reporting

Please select the one below that is the best fit for your research. If you are not sure, read the appropriate sections before making your selection.

☐ Life sciences ☐ Behavioural & social sciences ☒ Ecological, evolutionary & environmental sciences

For a reference copy of the document with all sections, see nature.com/documents/nr-reporting-summary-flat.pdf

Ecological, evolutionary & environmental sciences study design

All studies must disclose on these points even when the disclosure is negative.

Study description	We extracted phytoliths from the stomach content of a stem avialan from Jehol Biota and those phytoliths were recognized as angiosperm leaf which represent the earliest record of leaf feeding behavior in early birds.
Research sample	We sample two fragments close to each other in the gastric content of the fossil bird specimen.
Sampling strategy	We sampled twice for the fragments in gastric content. The two independant experiments are repeatable and both yield similar phytoliths.
Data collection	Phytolith extraction was conducted at the Key Laboratory of Vertebrate Evolution and Human Origins of Chinese Academy of Sciences, Institute of Vertebrate Palaeontology and Palaeoanthropology, Chinese Academy of Sciences by Wu Yan. Each phytolith was counted and photographed at 500 × magnification under the Nikon Eclipse LV100POL microscope By Wu Yan. Modern plants were collected from China National Botanical Garden South Garden, Gongga Mountain, Sichuan, Guangxi Province, China by Wuyan and Ge Yong. A phenom Pro X scanning electron microscope (SEM) with EDS was taken in Department of Archaeology and Anthropology, University of Chinese Academy Beijing, China by Wu yan.
Timing and spatial scale	The sampling were completed in 2019-2020 by the first and corresponding authors; in-lab experiments were completed in 2019-2022 by Wuyan.
Data exclusions	No data were excluded.
Reproducibility	Due to the nature of destructive sampling, we only sampled two fragments of the specimen and run two independent extraction experiments, both of which yield phytoliths; and therefore, the result is reproducible.
Randomization	N/A
Blinding	N/A

Did the study involve field work? ☐ Yes ☒ No

Reporting for specific materials, systems and methods

We require information from authors about some types of materials, experimental systems and methods used in many studies. Here, indicate whether each material, system or method listed is relevant to your study. If you are not sure if a list item applies to your research, read the appropriate section before selecting a response.

Materials & experimental systems

n/a	Involved in the study
☒	☐ Antibodies
☒	☐ Eukaryotic cell lines
☐	☒ Palaeontology and archaeology
☒	☐ Animals and other organisms
☒	☐ Clinical data
☒	☐ Dual use research of concern

Methods

n/a	Involved in the study
☒	☐ ChIP-seq
☒	☐ Flow cytometry
☒	☐ MRI-based neuroimaging

Palaeontology and Archaeology

Specimen provenance

Field trip issued by Ministry of Land and Resources of the People's Republic of China in 2006, and lead by Prof. Wang-Xiaolin and Prof. Zhou-zhonghe in IVPP. The new Jeholornis specimen described here was acquired from that trip in Jiufotang Formationin Gong-gao village, Western Liaoning province, northeastern China.

Specimen deposition

Specimen have been deposited in fossil collection of Institute of Vertebrate Paleontology and Paleoanthropology (Beijing, China) with a specimen number of IVPP V 14978 in 2006.

Dating methods

No new dating was used.

☒ Tick this box to confirm that the raw and calibrated dates are available in the paper or in Supplementary Information.

Ethics oversight

Destructive samplings of gastric contents follow the requirements and guidelines for Institute of Vertebrate Paleontology and Paleoanthropology. Permissions were acquired and granted by collection manager(Geng Binghe) as well as director (Deng Tao) of the institution (IVPP). The permission was obtained and Issued on 2019.

Note that full information on the approval of the study protocol must also be provided in the manuscript.
